# Supplementary material for: Outcomes of Different Haploidentical Transplantation Strategies from the Taiwan Blood and Marrow Transplantation Registry
Source: Cancers (Basel). 2022 Feb 21;14(4):1097. doi: 10.3390/cancers14041097 (PMC8870072; doi:10.3390/cancers14041097)
Supplement: Supplementary file 1 [file cancers-14-01097-s001.zip › cancers-1589940-supplementary.pdf]

Outcomes of different haploidentical transplantation strategies from the Taiwan Blood and Marrow Transplantation Registry

Xavier Cheng-Hong Tsai,[1,2,3] Tzu-Ting Chen,[4] Jyh-Pyng Gau,[5] Po-Nan Wang,[6] Yi-Chang Liu,[7,8] Ming-Yu Lien,[4] Chi-Cheng Li,[9,10] Ming Yao,[1,3] Bor-Sheng Ko[1,3]

[1]Division of Hematology, Department of Internal Medicine, National Taiwan University Hospital, Taipei, Taiwan

[2]Genome and Systems Biology Degree Program, National Taiwan University, Taipei, Taiwan

[3]Department of Hematological Oncology, National Taiwan University Cancer Center, Taipei, Taiwan

[4]Division of Hematology and Oncology, Department of Medicine, China Medical University Hospital, Taichung, Taiwan

[5]Division of Hematology, Department of Medicine, Taipei Veterans General Hospital, Taipei, Taiwan

[6]Division of Hematology, Department of Internal Medicine, Chang Gung Medical Foundation, Linkou Branch, Taoyuan, Taiwan

[7]Division of Hematology-Oncology, Department of Internal Medicine, Kaohsiung Medical University Hospital, Kaohsiung, Taiwan

[8]College of Medicine, Kaohsiung Medical University, Kaohsiung, Taiwan

[9]Tai-Cheng Cell Therapy Center, National Taiwan University, Taiwan

[10]Department of Hematology and Oncology, Buddhist Tzu-Chi General Hospital, Hualien, Taiwan.

Supplementary Table 2

Supplementary Figure Legend 4

Supplementary Figures..5

Supplementary Table S1. Post hoc test for CD34 dose

	Mean difference	Std. Error	P value
mGIAC vs PTCy with ATG	-2.51	0.56	<0.001
mGIAC vs PTCy without ATG	-3.39	0.67	<0.001
PTCy with ATG vs. PTCy without ATG	-0.87	0.76	0.489

Abbreviations: ATG, anti–thymocyte globulin

Supplementary Table S2. Comparison of clinical characteristics among patients with different haplo-HSCT approaches after propensity-score matching

Variables	Total (n = 129)	Modified GIAC (n = 86, 66.7%)	PTCy without ATG (n = 16, 12.4%)	PTCy with ATG (n = 27, 20.9%)	P value
**Sex** $^{\alpha}$					0.341
Male	62 (48.1%)	40 (46.5%)	6 (37.5%)	16 (59.3%)	
Female	67 (51.9%)	46 (53.5%)	10 (62.5%)	11 (40.7%)	
**Age, years** $^{\beta}$	42.1 (18.7-69.2)	42.0 (18.7-69.2)	42.0 (21.8-63.7)	44.9 (18.9-68.3)	0.700
**Disease** $^{\alpha}$					
AML	70 (54.3%)	51 (59.3%)	5 (31.3%)	14 (51.9%)	0.113
MDS	9 (7.0%)	7 (8.1%)	0 (0%)	2 (7.4%)	0.500
MDS/MPN	4 (3.1%)	1 (1.2%)	2 (12.5%)	1 (3.7%)	0.055
ALL	26 (20.2%)	18 (20.9%)	3 (18.8%)	5 (18.5%)	0.953
MPAL	2 (1.6%)	2 (2.3%)	0 (0%)	0 (0%)	0.602
CML	4 (3.1%)	1 (1.2%)	1 (6.3%)	2 (7.4%)	0.195
NHL	10 (7.8%)	3 (3.5%)	5 (31.3%)	2 (7.4%)	0.001
HL	3 (2.3%)	2 (2.3%)	0 (0%)	1 (3.7%)	0.738
Myeloma	1 (0.8%)	1 (1.2%)	0 (0%)	0 (0%)	0.777
**Conditioning** $^{\alpha}$					0.833
Myeloablative	28 (21.7%)	20 (23.3%)	3 (18.8%)	5 (18.5%)	
Reduced intensity	101 (78.3%)	66 (76.7%)	13 (81.3%)	22 (81.5%)	
**ATG dose per kilogram** $^{\gamma}$	6.0 (2.0-7.5)	6.0 (5.0-7.5)	0	4.0 (2.0-7.5)	<0.001
**Stem cell source** $^{\alpha}$					<0.001
BM + mobilized PB	86 (66.7%)	86 (100%)	0 (0%)	0 (0%)	
Mobilized PB	43 (33.3%)	0 (0%)	16 (100%)	27 (100%)	
**Donor relationship** $^{\alpha}$					0.584
Child	53 (41.1%)	35 (40.7%)	7 (43.8%)	11 (40.7%)	
Parent	37 (28.7%)	27 (31.4%)	2 (12.5%)	8 (29.6%)	
Sibling	39 (30.2%)	7 (27.9%)	7 (43.8%)	8 (29.6%)	
**Donor–recipient sex combination** $^{\alpha}$					0.736
Female donor to male recipient	33 (25.6%)	22 (25.6%)	3 (18.8%)	8 (29.6%)	
Other combinations	96 (74.4%)	64 (74.4%)	13 (81.2%)	19 (70.4%)	
**Recipient CMV serostatus** $^{\alpha\delta}$					0.898
Negative	11 (8.5%)	8 (9.3%)	1 (9.1%)	2 (18.2%)	
Positive	118 (91.5%)	78 (90.7%)	15 (93.8%)	25 (92.6%)	
Missing	1 (0.8%)	0 (0%)	0 (0%)	1 (3.7%)	
**CD34 (10^6/kg)** $^{\gamma\epsilon}$	5.1 (1.3-21.2)	5.0 (2.4-8.3)	5.3 (3.0-13.5)	5.9 (1.3-21.2)	
**Disease Risk Index** $^{\alpha}$					0.136
Low	8 (6.2%)	5 (5.8%)	0 (0%)	3 (11.1%)	
Intermediate	52 (40.3%)	32 (37.2%)	6 (37.5%)	14 (51.9%)	
High	56 (43.4%)	40 (46.5%)	10 (62.5%)	5 (22.2%)	
Very high	13 (10.1%)	9 (10.5%)	0 (0%)	4 (14.8%)	
**Year of HSCT**	2016 (2011-2019)	2016 (2013-2019)	2016 (2014-2019)	2017 (2011-2019)	

Abbreviations: ALL, acute lymphoblastic leukemia; AML, acute myeloid leukemia; ATG, anti–thymocyte globulin; BM, bone marrow; CMV, cytomegalovirus; CR, complete remission; HL, Hodgkin lymphoma; MDS, myelodysplastic syndrome; MPAL, mixed phenotypic acute leukemia; NHL, non–Hodgkin lymphoma; PB, peripheral blood
α Number of patients (%)
β Mean (range)
γ Median (range)
δ Based on patients with available data
ε Combination of bone marrow and peripheral stem cell doses

Supplementary Table S3. Causes of death among patients receiving different haplo-HSCT

strategies

Cause of death	Modified GIAC (n = 110)	PTCy without ATG (n = 26)	PTCy with ATG (n = 42)	P value
Overall	54	20	23	
Relapse	32 (59.3%)	10 (50%)	12 (52.2%)	0.620
Infection	12 (22.2%)	5 (25%)	8 (34.8%)	0.309
GvHD	5 (9.3%)	2 (10%)	1 (4.3%)	0.589
Graft failure	2 (3.7%)	2 (10%)	0 (%)	0.102
Interstitial pneumonia	1 (1.9%)	0 (%)	1 (4.3%)	0.625
Intracerebral hemorrhage	0 (0%)	1 (5%)	0 (0%)	0.053
Other	2 (3.7%)	0 (%)	1 (4.3%)	0.748

Supplementary Figure S1. Modified GIAC protocol for myeloablative conditioning (A) and reduced intensity conditioning (B).

Supplementary Figure S2. The outcome analyses of patients receiving haplo–HSCT after propensity score matching, including cumulative incidence of relapse (A), nonrelapse mortality (B), overall survival (C), and GvHD/relapse–free survival (D).

Supplementary Figure S3. (A) The univariate analysis of cumulative incidence of relapse (CIR) yielded that advanced disease status and grade III-IV acute GvHD were significant prognostic factors. (B) In the univariate analysis of nonrelapse mortality (NRM), the variables had no prognostic impact. (C) The univariate analysis of GvHD/relapse-free survival (GRFS) yielded advanced disease status, grade III-IV acute GvHD, and extensive chronic GvHD were significant prognostic factors. (D) The univariate analysis of overall survival (OS) yielded advanced disease status, grade III-IV acute GvHD, extensive chronic GvHD, stem cell source, and recipient CMV serostatus were significant prognostic factors.

Supplementary Figure S4. Comparison of nonrelapse mortality (A) and cumulative incidence of relapse (B) in patients with low/intermediate-risk diseases receiving haplo-HSCT.

Supplementary Figure S5. Comparison of cumulative incidence of relapse (A) and nonrelapse mortality (B) in patients with high/very-high-risk diseases receiving haplo-HSCT.

Supplementary Figure S1A

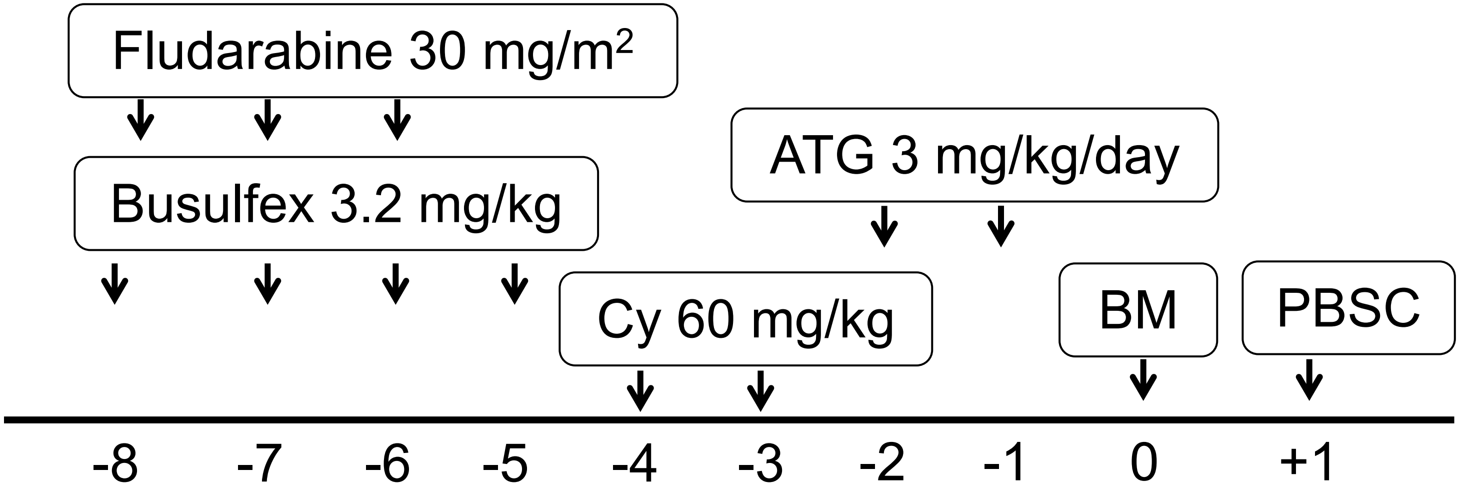

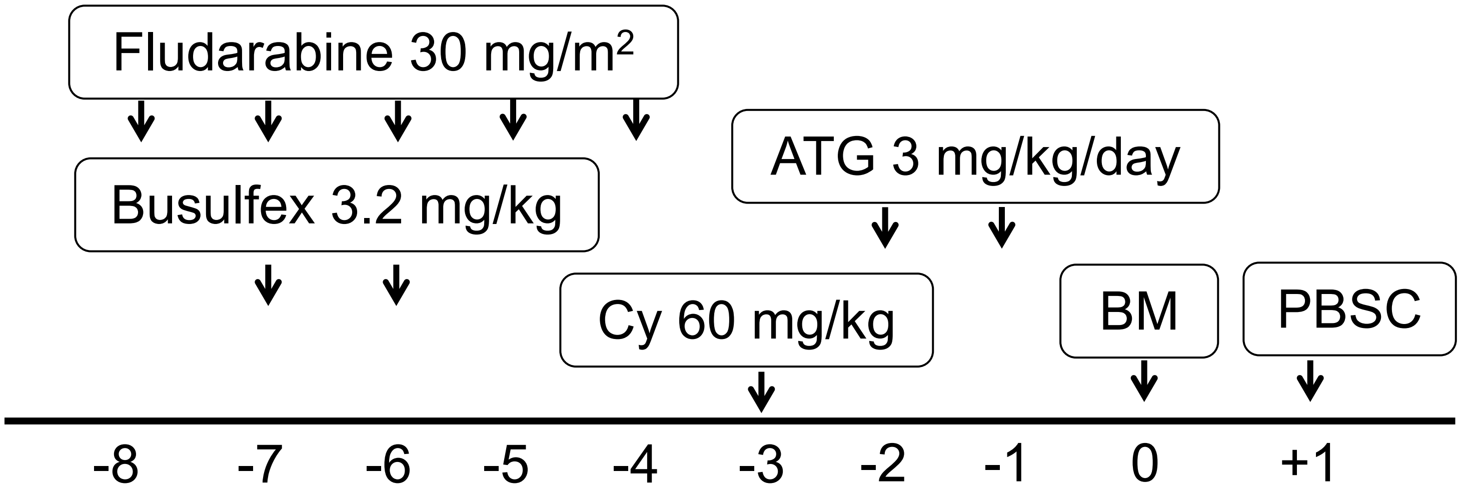
Fludarabine 30 mg/m2
Busulfex 3.2 mg/kg
ATG 3 mg/kg/day
Cy 60 mg/kg
BM
PBSC
-8
-7
-6
-5
-4
-3
-2
-1
0
+1

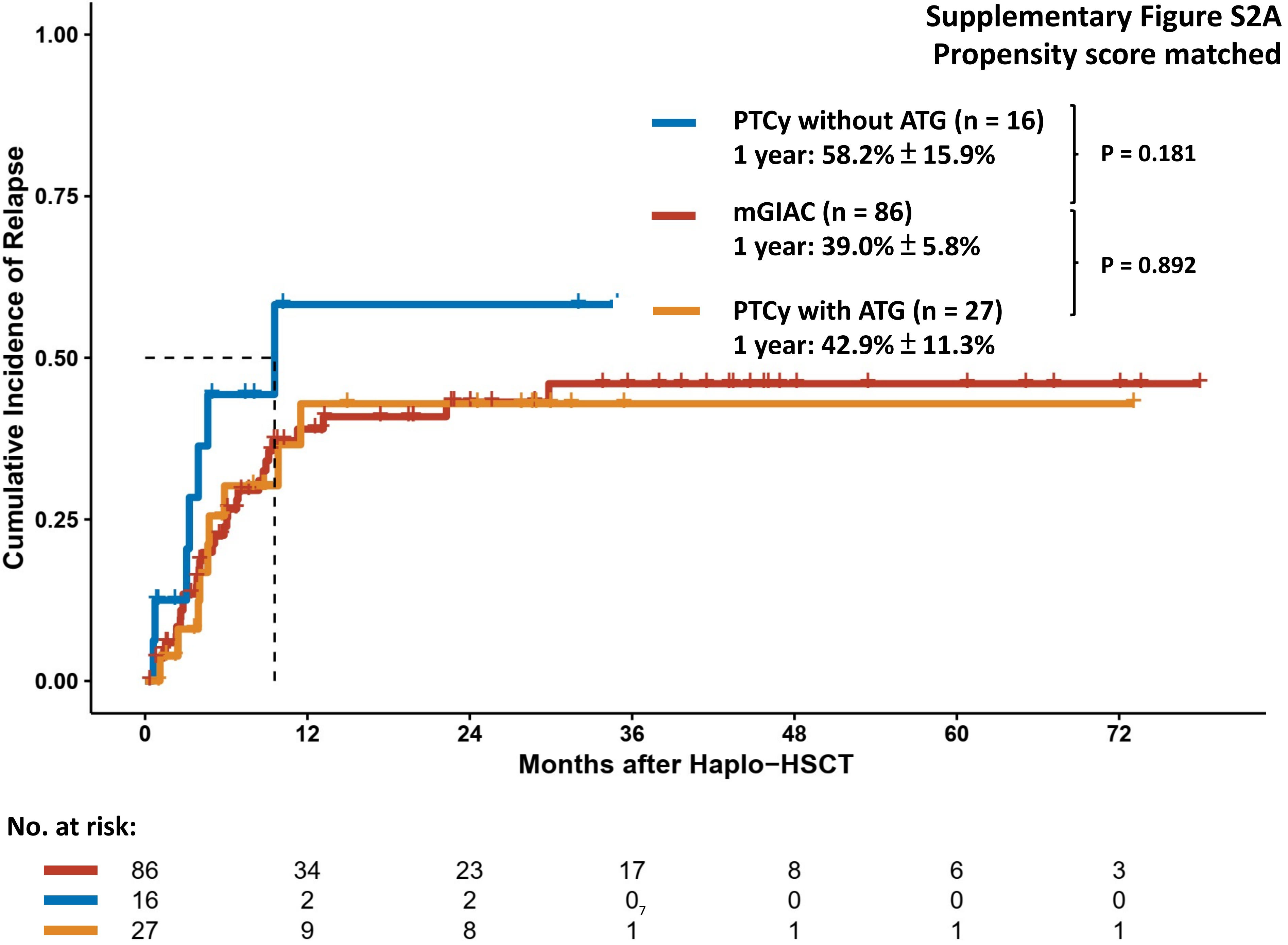
Supplementary Figure S2A
Propensity score matched
PTCy without ATG (n = 16)
1 year: 58.2% ± 15.9%
mGIAC (n = 86)
1 year: 39.0% ± 5.8%
PTCy with ATG (n = 27)
1 year: 42.9% ± 11.3%
P = 0.181
P = 0.892
Cumulative Incidence of Relapse
1.00
0.75
0.50
0.25
0.00
0
12
24
36
48
60
72
Months after Haplo−HSCT
No. at risk:
86 34 23 17 8 6 3
16 2 2 0 0 0 0
27 9 8 1 1 1 1

Supplementary Figure S2B
Propensity score matched

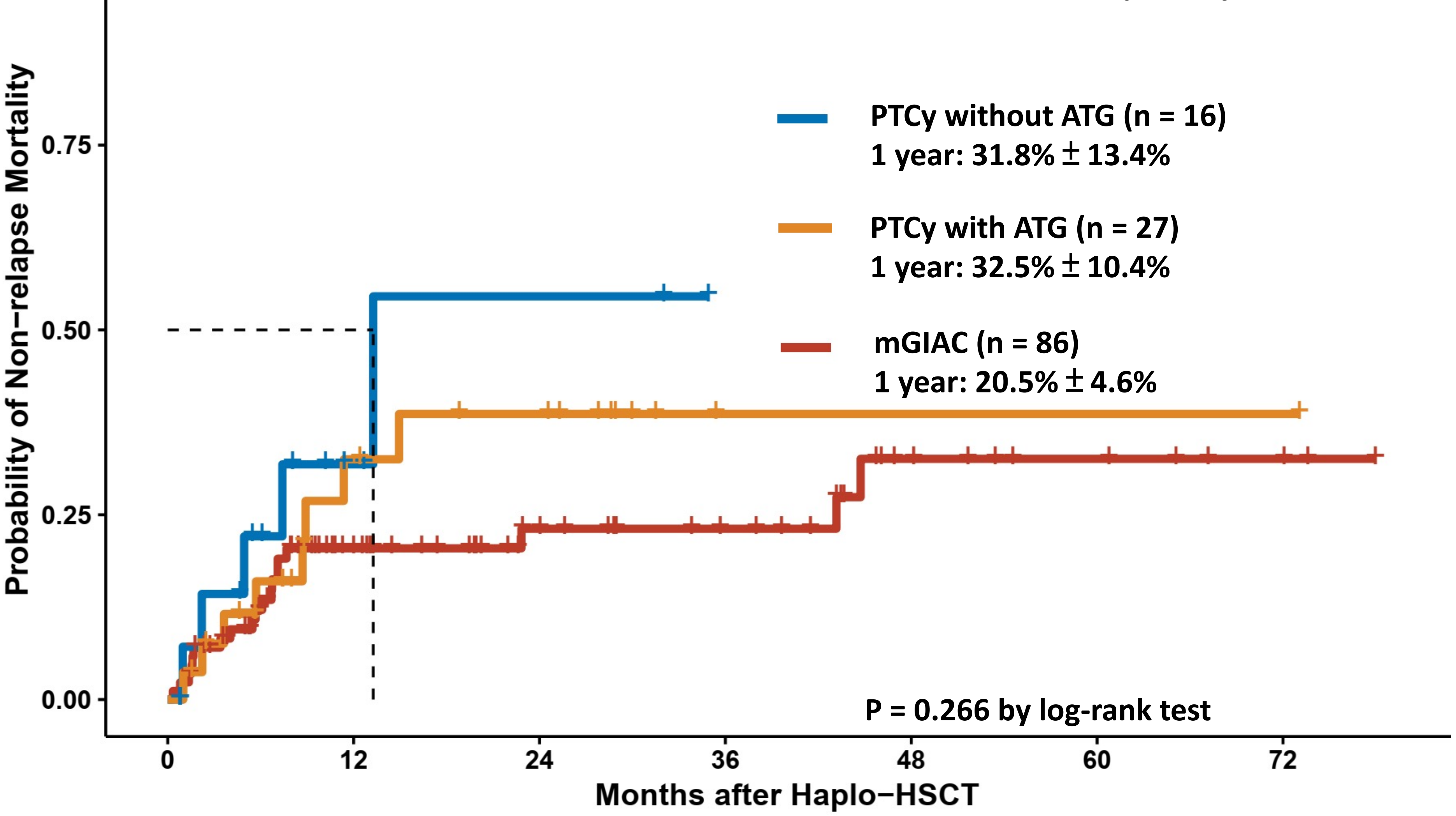

No. at risk:

mGIAC	86	45	28	21	10	6	3
PTCy without ATG	16	4	2	0	0	0	0
PTCy with ATG	27	12	9	1	1	1	1

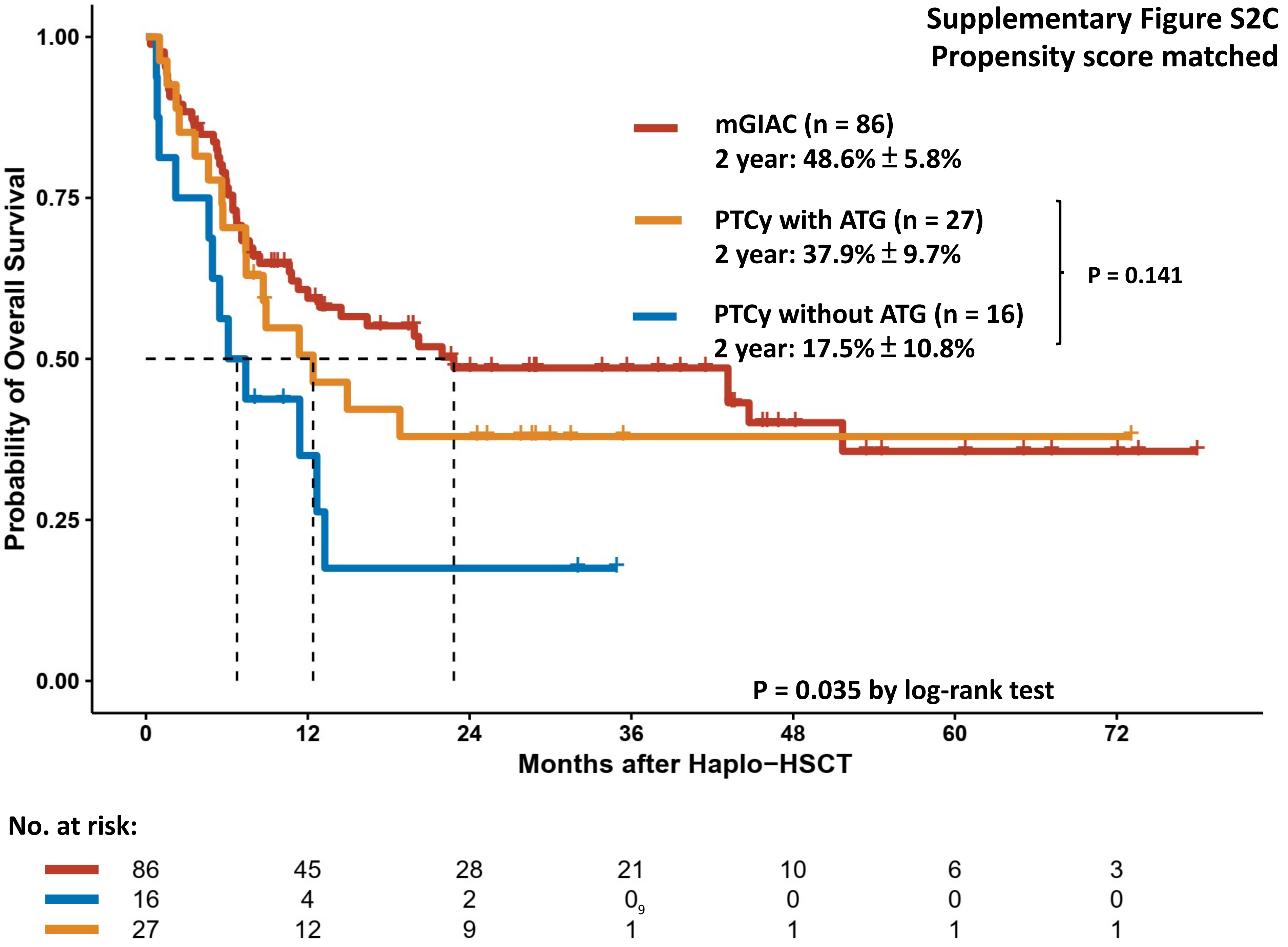

Supplementary Figure S2C
Propensity score matched
mGIAC (n = 86)
2 year: 48.6% ± 5.8%
PTCy with ATG (n = 27)
2 year: 37.9% ± 9.7%
PTCy without ATG (n = 16)
2 year: 17.5% ± 10.8%
P = 0.141
P = 0.035 by log-rank test
Probability of Overall Survival
1.00
0.75
0.50
0.25
0.00
0
12
24
36
48
60
72
Months after Haplo−HSCT
No. at risk:
86 45 28 21 10 6 3
16 4 2 0 0 0 0
27 12 9 1 1 1 1

Supplementary Figure S2D
Propensity score matched

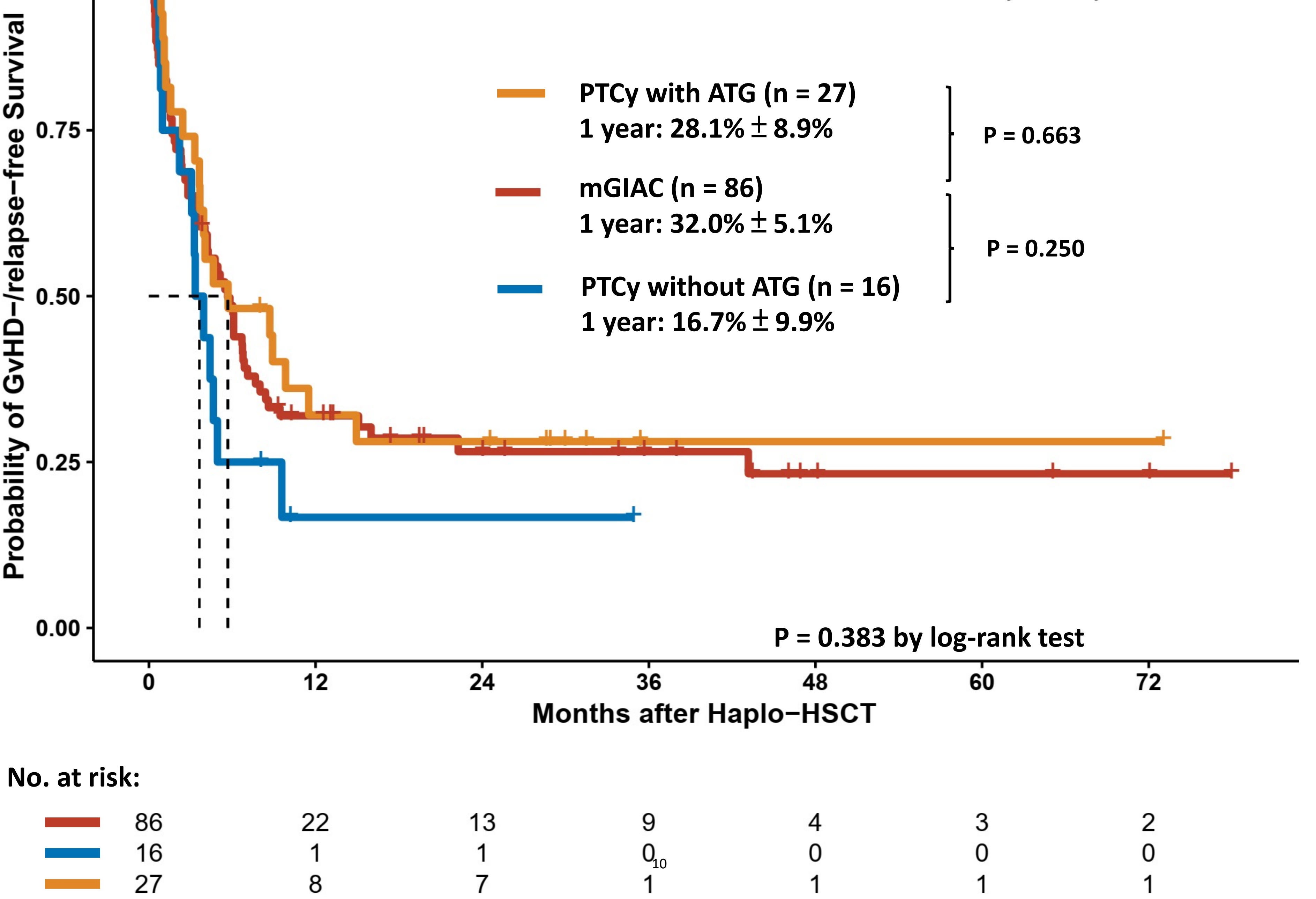

Supplementary Figure S3A

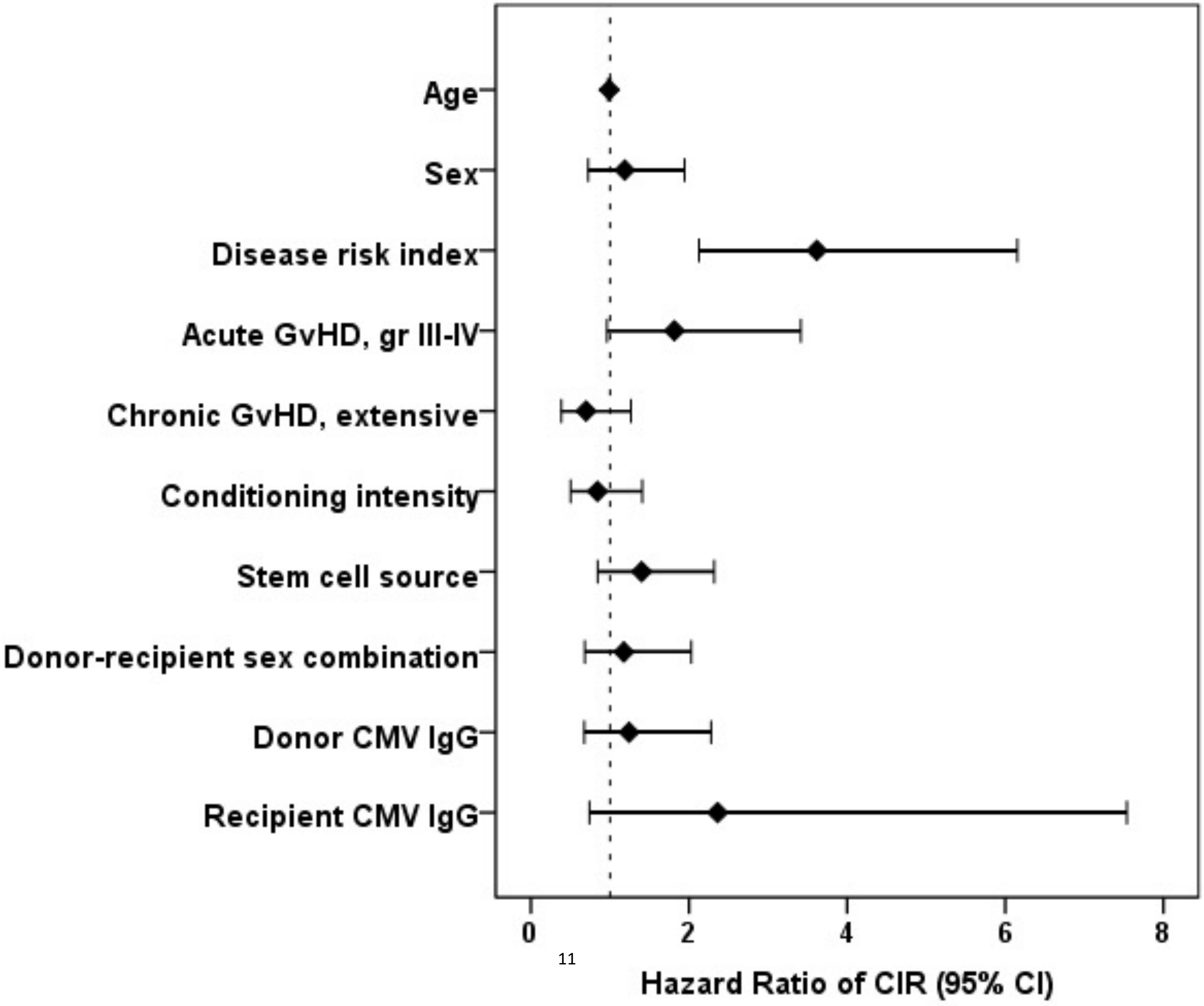

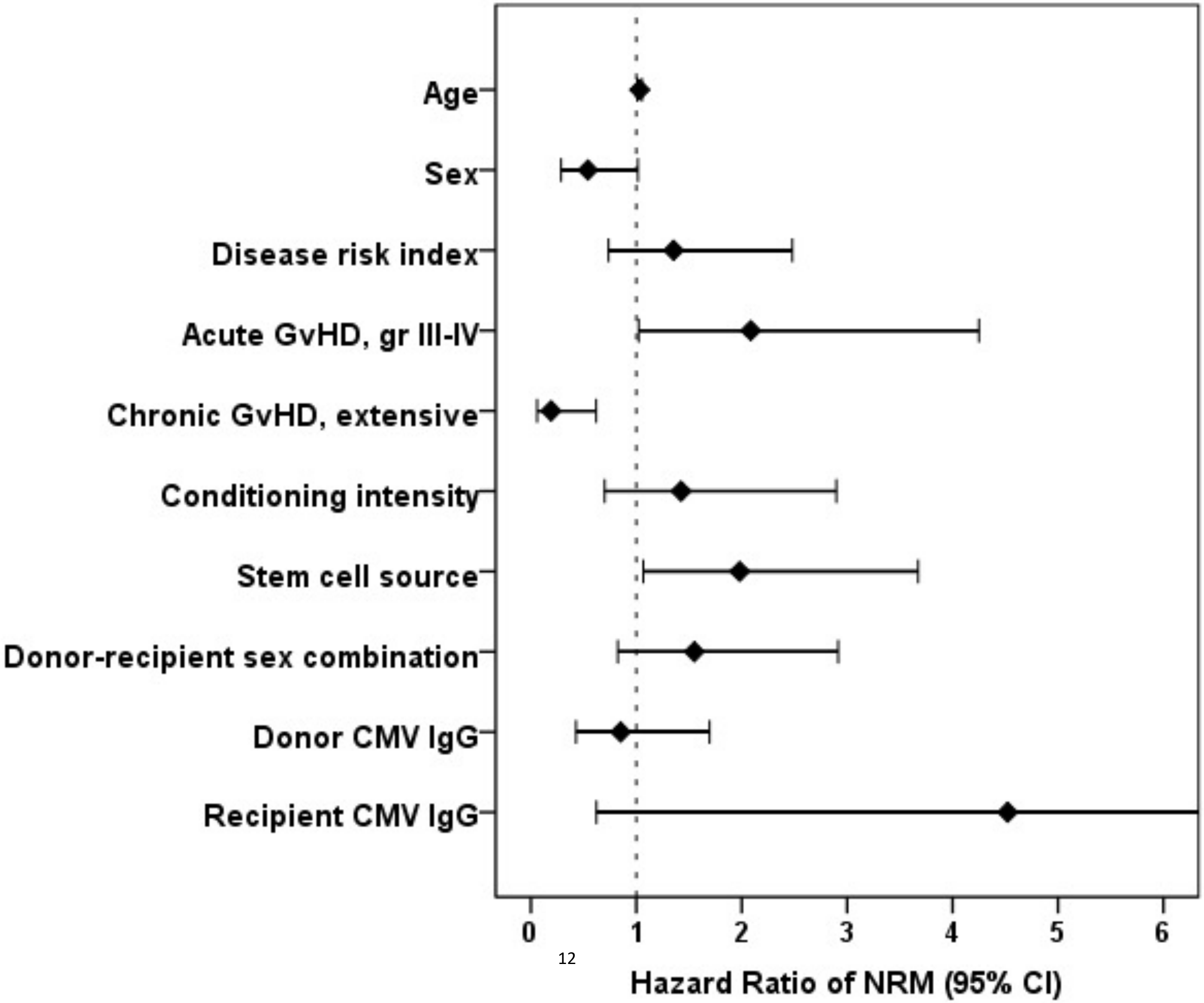
Age
Sex
Disease risk index
Acute GvHD, gr III-IV
Chronic GvHD, extensive
Conditioning intensity
Stem cell source
Donor-recipient sex combination
Donor CMV IgG
Recipient CMV IgG
0
1
2
3
4
5
6
Hazard Ratio of NRM (95% CI)

Supplementary Figure S3C

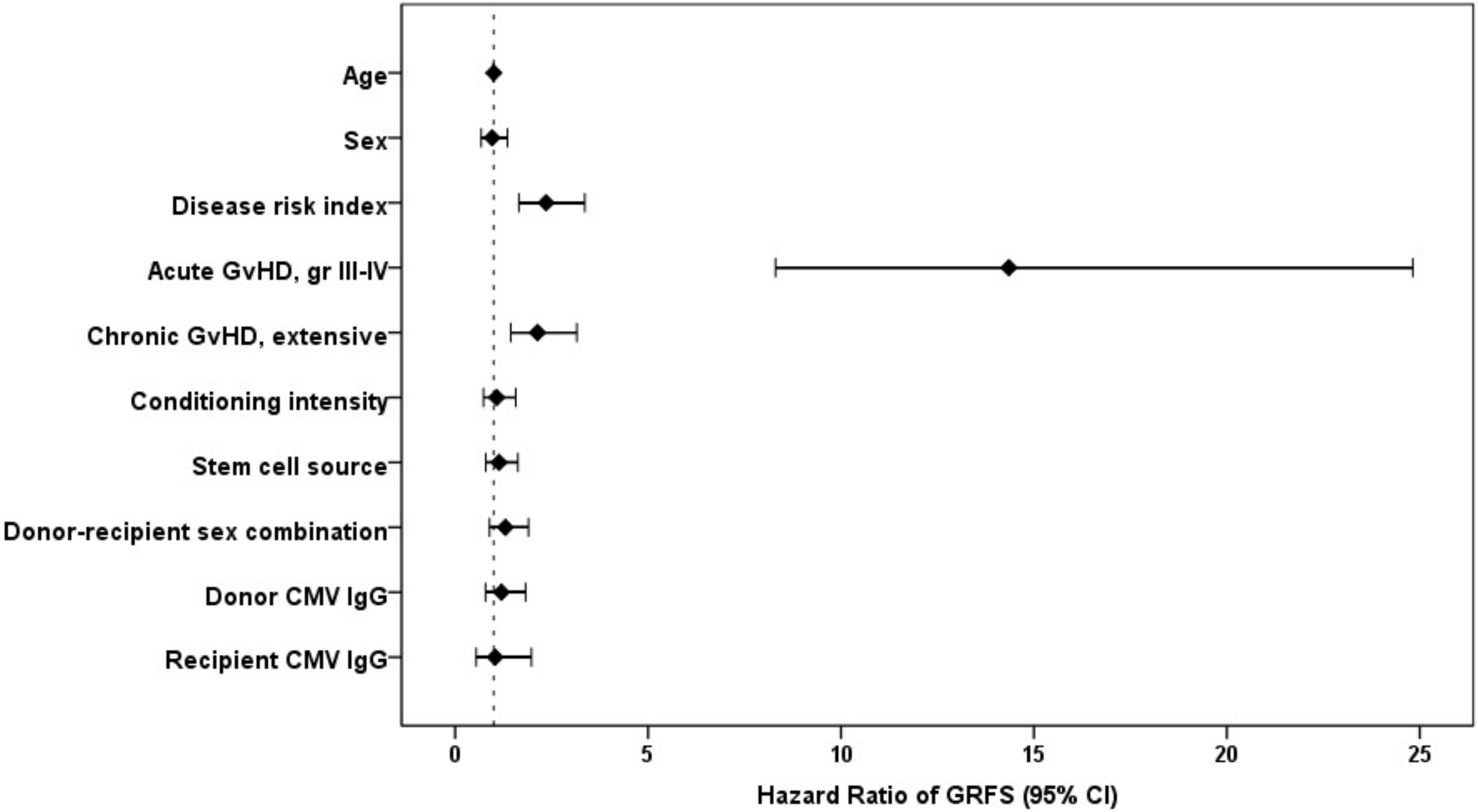

Supplementary Figure S3D

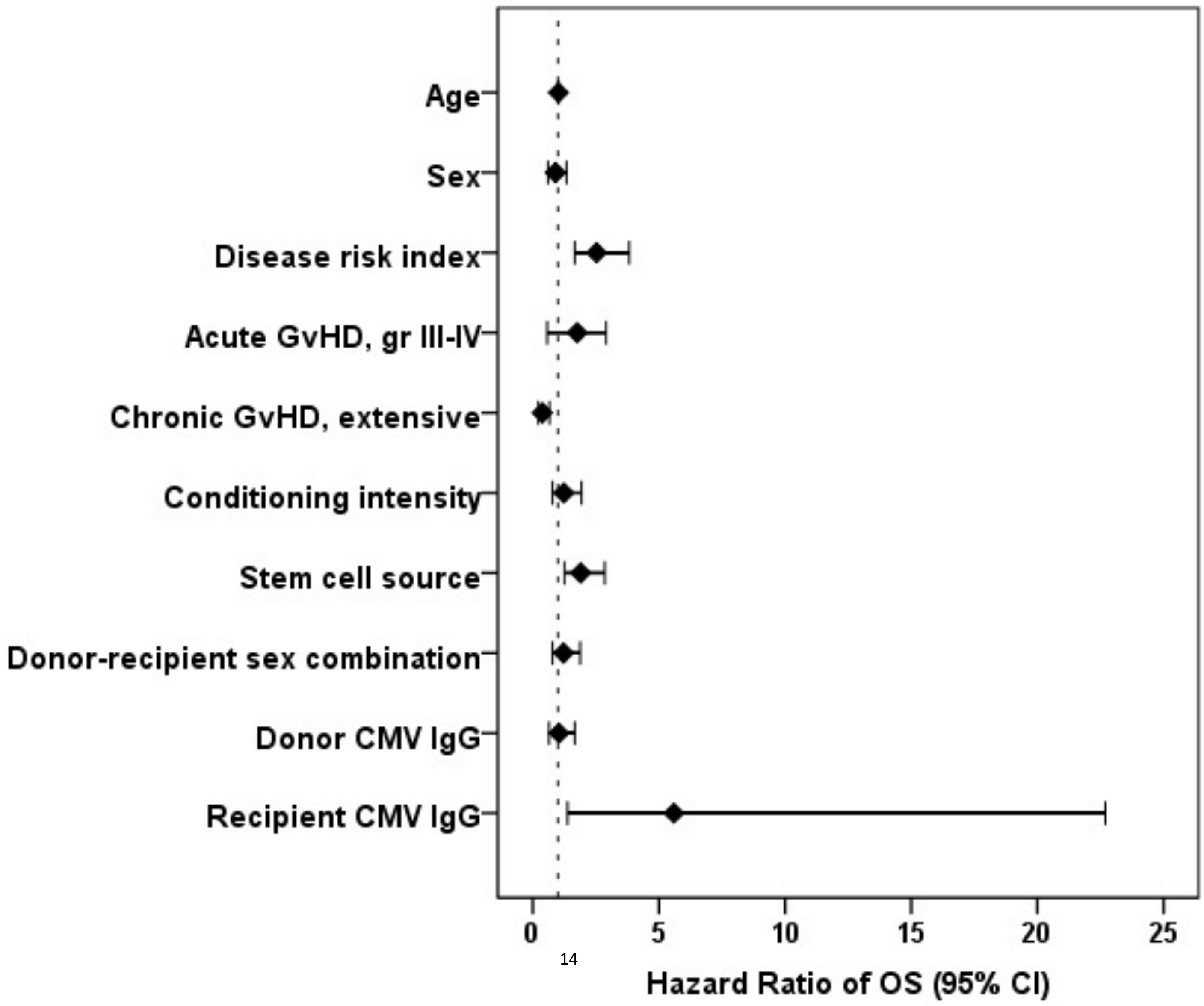

Supplementary Figure S4A
Low/Intermediate-risk

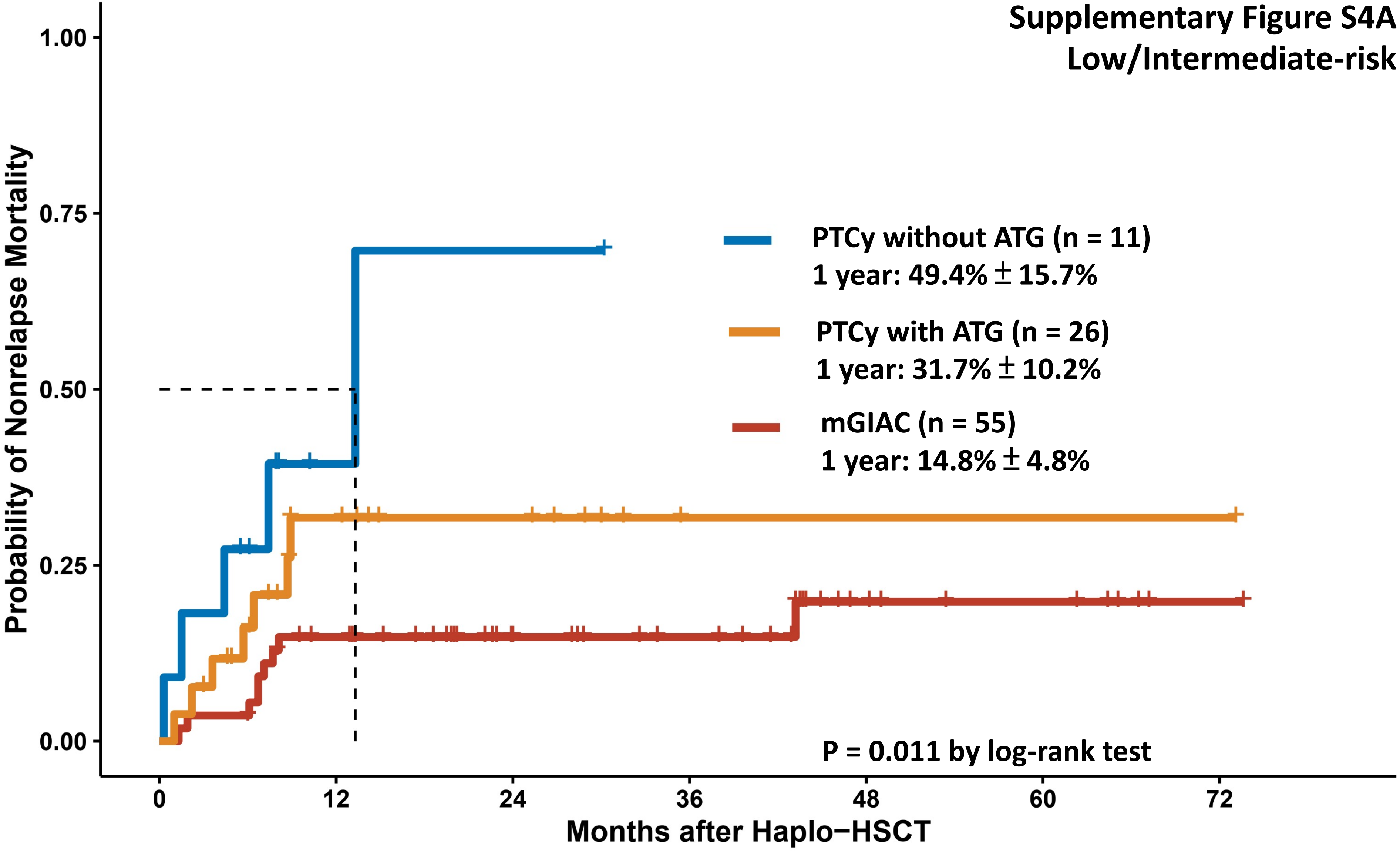

No. at risk:

	55	42	27	21	9	6	1
	11	2	1	0	0	0	0
	26	11	7	1	1	1	1

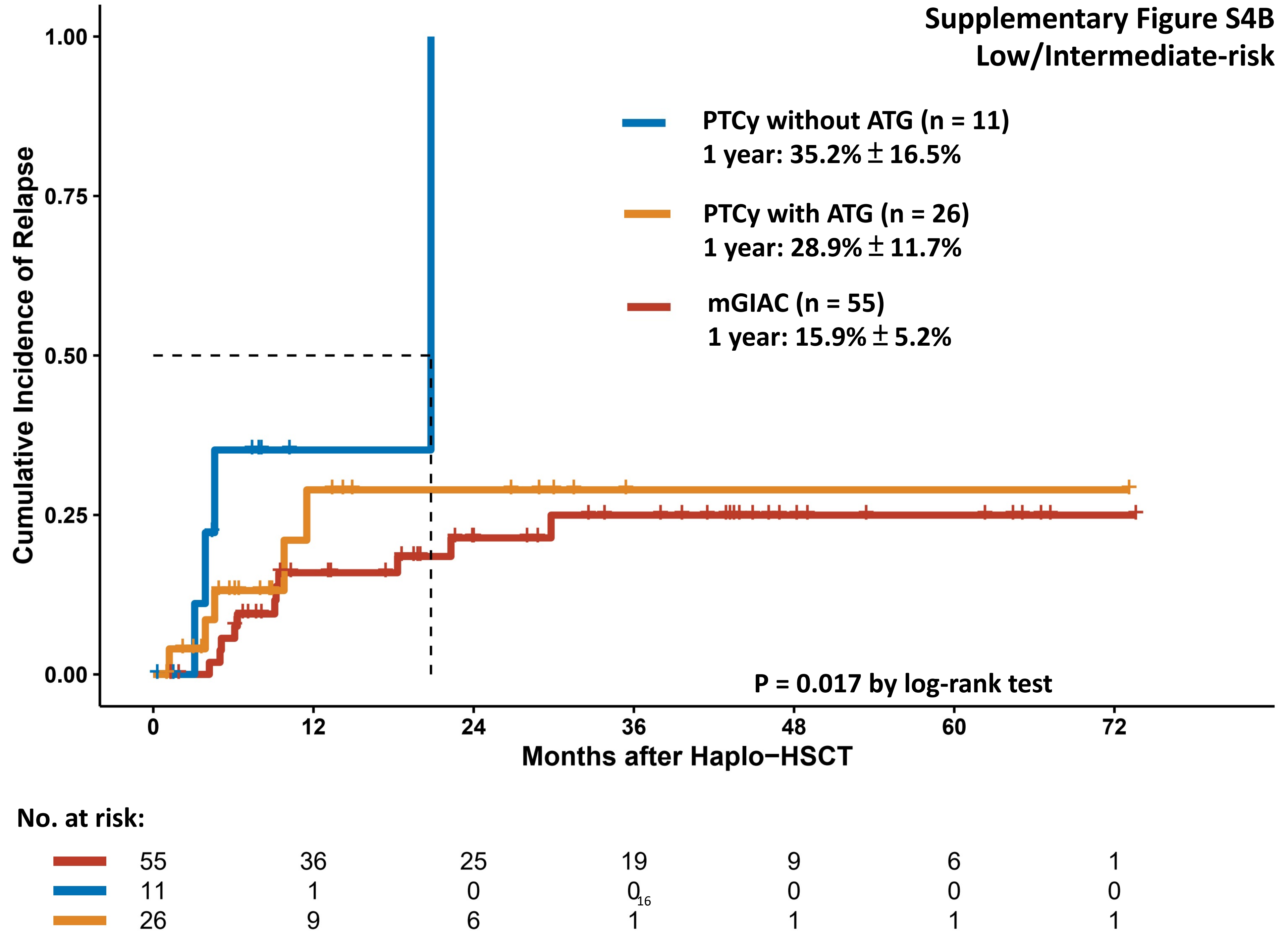

Supplementary Figure S4B
Low/Intermediate-risk
PTCy without ATG (n = 11)
1 year: 35.2% ± 16.5%
PTCy with ATG (n = 26)
1 year: 28.9% ± 11.7%
mGIAC (n = 55)
1 year: 15.9% ± 5.2%
Cumulative Incidence of Relapse
1.00
0.75
0.50
0.25
0.00
P = 0.017 by log-rank test
0
12
24
36
48
60
72
Months after Haplo−HSCT
No. at risk:
55 36 25 19 9 6 1
11 1 0 0 0 0 0
26 9 6 1 1 1 1

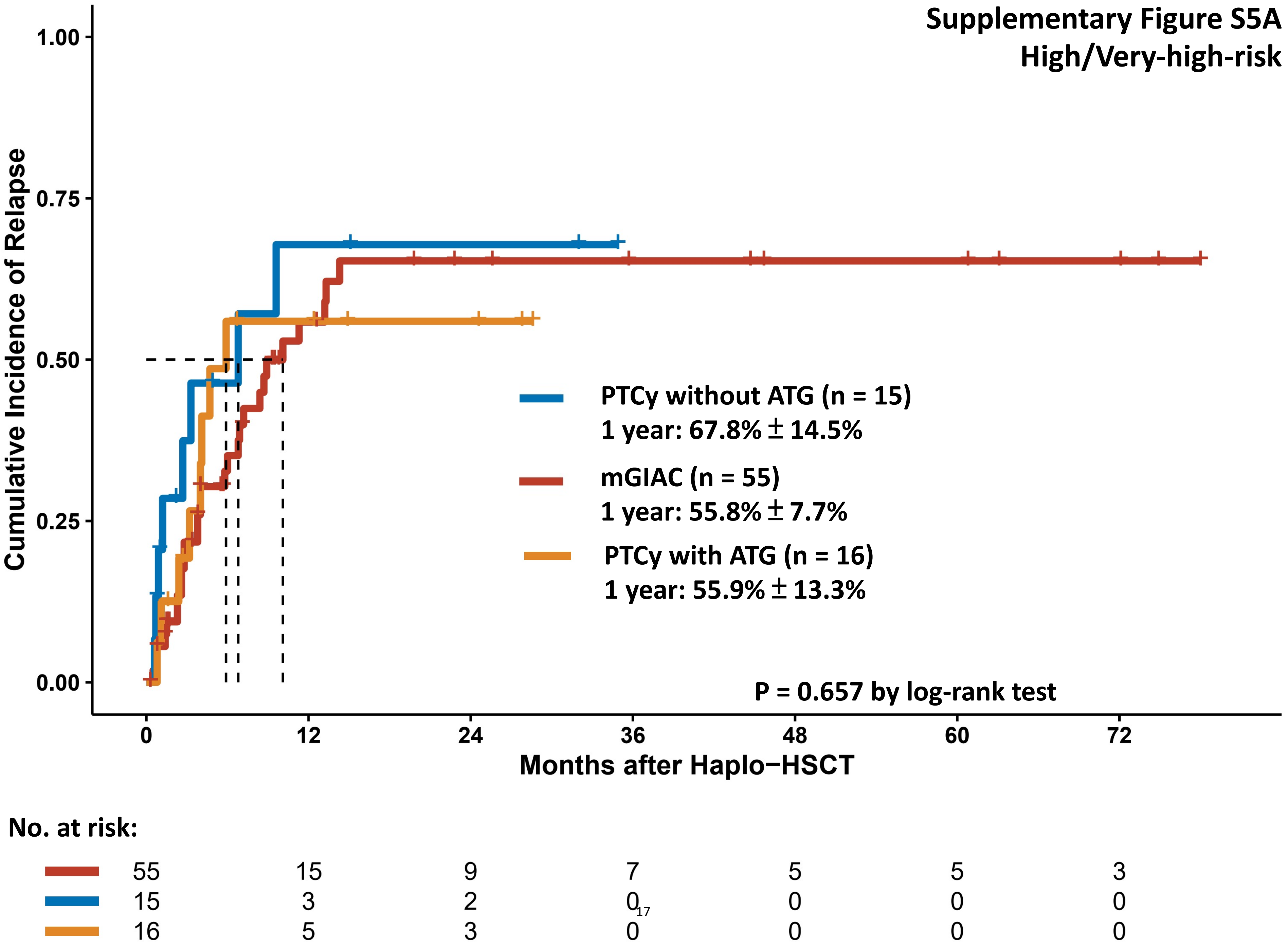
Supplementary Figure S5A
High/Very-high-risk
Cumulative Incidence of Relapse
1.00
0.75
0.50
0.25
0.00
0
12
24
36
48
60
72
Months after Haplo−HSCT
PTCy without ATG (n = 15)
1 year: 67.8% ± 14.5%
mGIAC (n = 55)
1 year: 55.8% ± 7.7%
PTCy with ATG (n = 16)
1 year: 55.9% ± 13.3%
P = 0.657 by log-rank test
No. at risk:
55 15 9 7 5 5 3
15 3 2 0 0 0 0
16 5 3 0 0 0 0

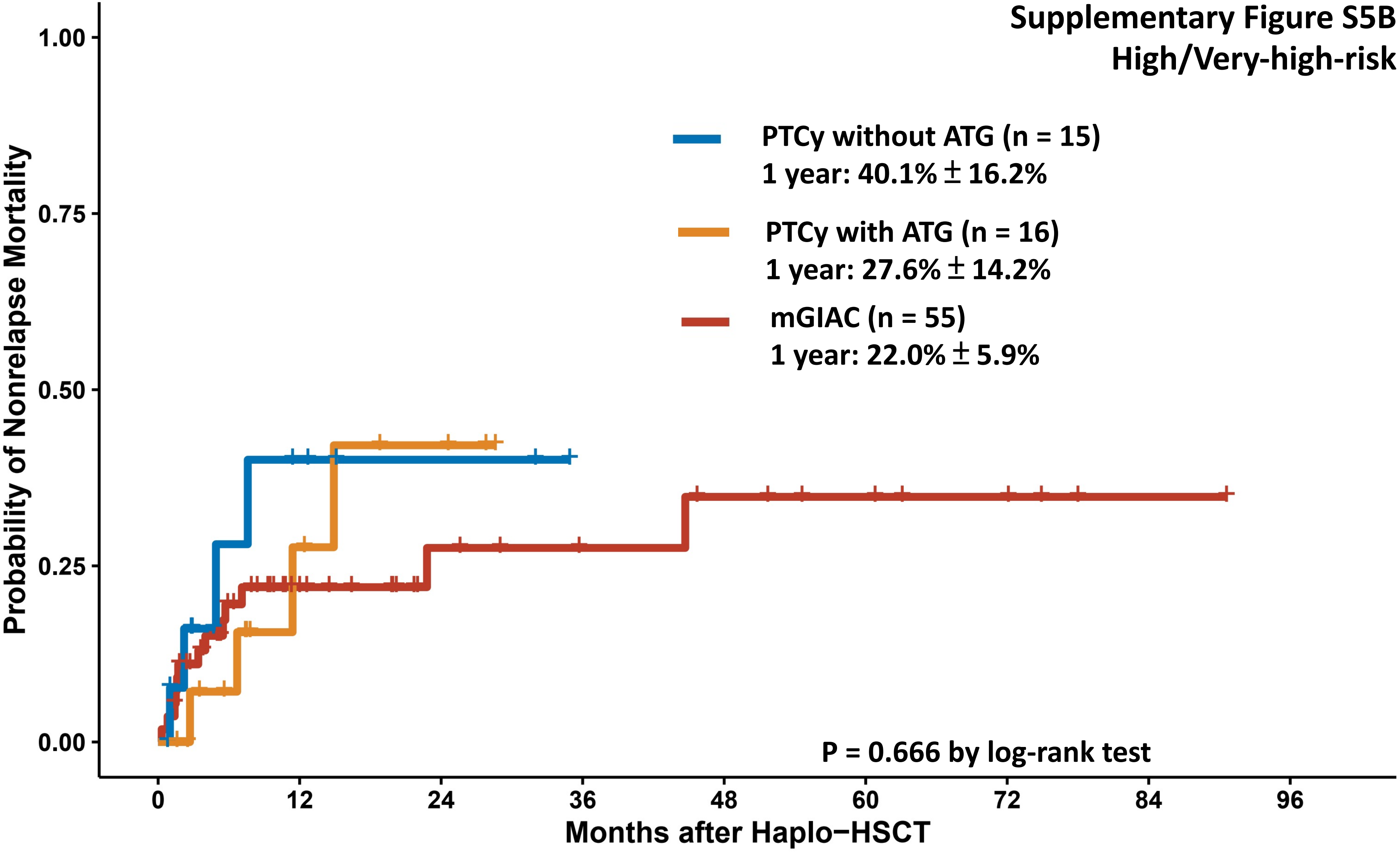

No. at risk:

mGIAC	55	24	13	10	8	6	4	1	0
PTCy without ATG	15	4	2	0	0	0	0	0	0
PTCy with ATG	16	6	3	0	0	0	0	0	0
